# Supplementary material for: Inactivation of Sirt6 ameliorates muscular dystrophy in mdx mice by releasing suppression of utrophin expression
Source: Nat Commun. 2022 Jul 20;13:4184. doi: 10.1038/s41467-022-31798-z (PMC9300598; doi:10.1038/s41467-022-31798-z)
Supplement: Supplementary file 1 — Supplementary Information [file 41467_2022_31798_MOESM1_ESM.pdf]

Supplementary Information

Inactivation of *Sirt6* ameliorates muscular dystrophy in *mdx* mice by releasing suppression of utrophin expression

Angelina M. Georgieva[1], Xinyue Guo[1], Marek Bartkuhn[2], Stefan Günther[1], Carsten Künne[1], Christian Smolka[1], Ann Atzberger[1], Ulrich Gärtner[3], Kamel Mamchaoui[4], Eva Bober[1], Yonggang Zhou[1], Xuejun Yuan[1]*, Thomas Braun[1]*

[1]Department of Cardiac Development and Remodeling, Max Planck Institute for Heart and Lung Research, 61231, Bad Nauheim, Germany

[2]Biomedical Informatics and Systems Medicine, Justus Liebig University, Giessen, Germany

[3]Institute for Anatomy and Cell Biology, University of Giessen, Giessen, Germany

[4]Sorbonne Université, Inserm, Institut de Myologie, Centre de Recherche en Myologie, F-75013 Paris, France.

*Corresponding authors: Xuejun Yuan and Thomas Braun; xuejun.yuan@mpi-bn.mpg.de, thomas.braun@mpi-bn.mpg.de

Supplementary Information **Page**

Supplementary Information	Page
Supplementary Figure 1	3
Supplementary Figure 2	4
Supplementary Figure 3	6
Supplementary Figure 4	8
Supplementary Figure 5	10
Supplementary Figure 6	11
Supplementary Figure 7	12
Supplementary Figure 8	13
Supplementary Figure 9	14
Supplementary Figure 10	15
Supplementary Table 1	16
Supplementary Table 2	17
Supplementary Table 3	18
Supplementary Table 4	19
Supplementary Table 5	20

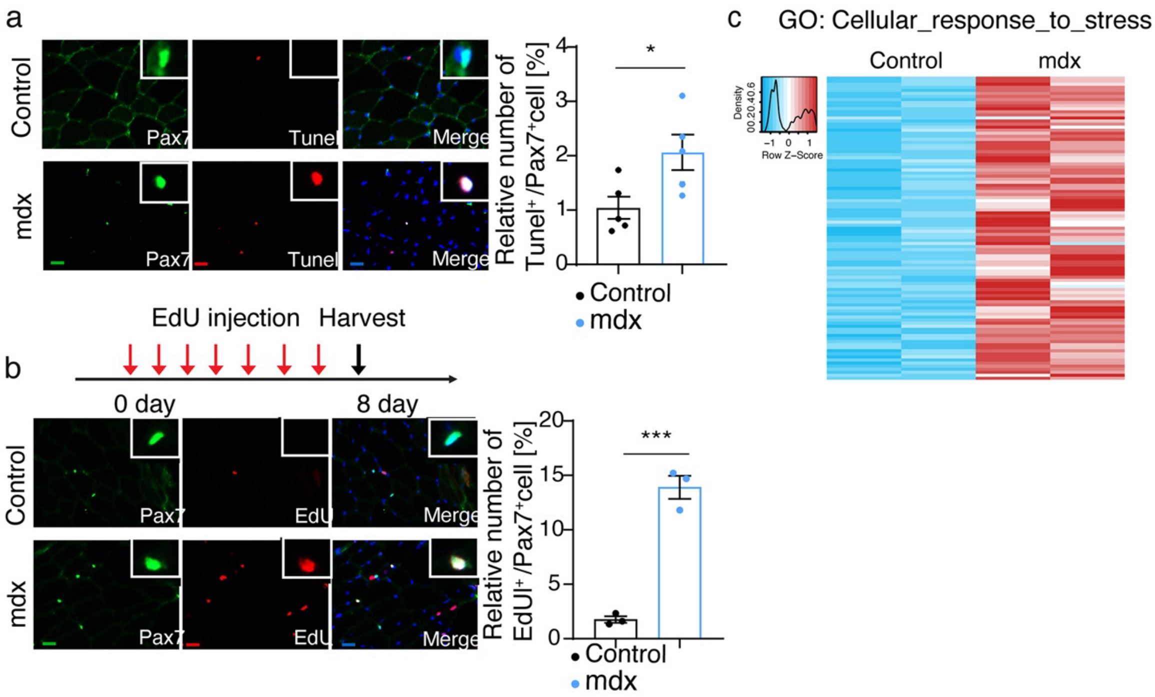

Supplementary Figure 1. *Mdx* MuSCs show increased apoptosis and proliferation a TUNEL assay of $PAX7^+$ MuSCs in TA muscles of control and *mdx* mice. Scale bar: 20μm. Relative numbers of $Tunel^+/PAX7^+$ MuSCs are shown on the right. (Unpaired two-tailed t-test: *p=0.0319; n=5 for each group). **b** EdU incorporation in $PAX7^+$ MuSCs of TA muscles from control and *mdx* mice. Scale bar: 20μm. Quantifications are shown on the right (Unpaired two-tailed t-test: ***p=0.0004; n=3 for each group). **c** Heat map of RNA-seq data visualizing upregulation of genes involved in cellular responses to stress [http://www.informatics.jax.org/go/term/GO:0033554] in *mdx* compared to the control MuSCs (n=2 for each group). A list of gene names shown in the heatmap is provided in Supplementary table 1. 12-weeks old male mice were used. Data are presented as mean ± SEM (a-b). Source data are provided in the Source Data file.

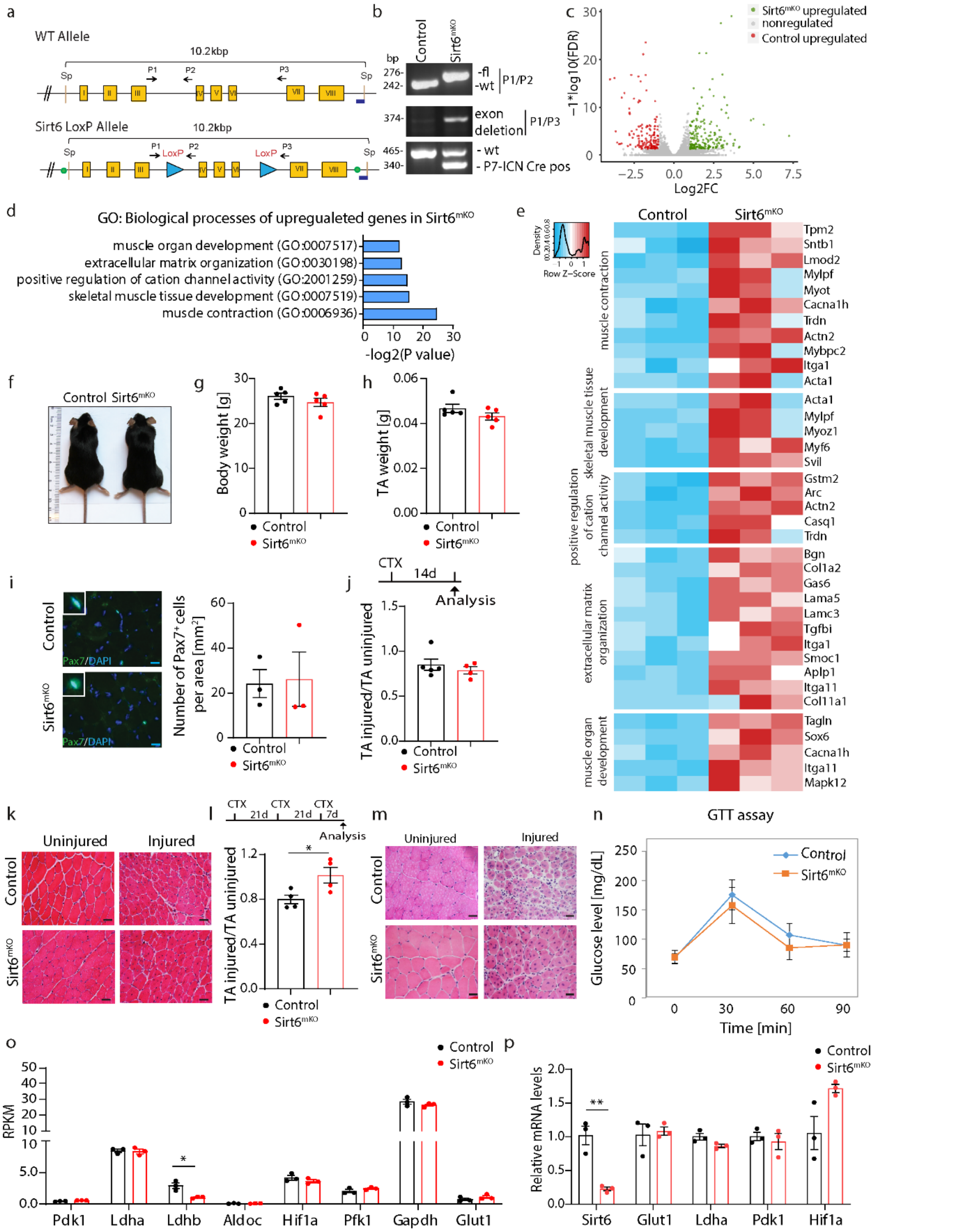

Supplementary Figure 2. ***Sirt6* is dispensable for skeletal muscle development and skeletal muscle homeostasis. a** Schematic representation of wild-type and conditional alleles of the *Sirt6* gene. Primers for genotyping are indicated by black arrows. P1/P2 primers were used to detected wild-type and floxed allele. P1/P3 primers were used to detect the deleted allele. **b** Genotyping by PCR to detect wild-type, floxed and deleted alleles in FACS-sorted MuSCs. **c** Volcano plot of differentially expressed genes in Sirt6^{mKO} compared to freshly isolated control MuSCs. **d** Top 5 biological processes (BP) upregulated in *Sirt6^{mKO}* versus wild type MuSCs. **e** Heat map of RNA-seq data visualizing upregulation of genes in *Sirt6^{mKO}* MuSCs contained in

top 5 GO-terms (n=3). Benjamini-Hochberg test, two-sided. **f** Images of 12-week-old control (Sirt6$^{fl/fl}$ Pax7ICN^{neg}) and *Sirt6^{mKO}* (Sirt6$^{fl/fl}$ Pax7ICN^{pos}) mice. **g** Body weight of 12-week-old control and *Sirt6^{mKO}* male mice (Unpaired two-tailed t-test: ns p >0.05; n=6). **h** TA muscle weight of 12-weeks-old control and *Sirt6^{mKO}* male mice (Unpaired two-tailed t-test: ns p >0.05; n=5 for each group). **i** Immunofluorescence staining of PAX7+ positive MuSCs in control and *Sirt6^{mKO}* TA muscle cryo-sections. Scale bar: 20 µm. Numbers of MuSC per mm^2 section area are shown in the right panel (n=3). **j** Ratios of TA muscle masses after CTX injection compared to untreated samples dissected from control (n=5) or *Sirt6^{mKO}* mice (n=4) (Unpaired two-tailed t-test ns: p>0.05). Top panel: scheme to induce muscle regeneration in control and Sirt6^{mKO} littermates. **k** H&E staining of TA muscles in control (n=5) and Sirt6^{mKO} (n=4) mice after muscle injury. Scale bar: 20 µm. **l** Schematic outline of the repeated muscle regeneration assay. Ratios of TA muscle masses with and without CTX injection are shown (Unpaired two-tailed t-test: *p =0.0389 n = 4 for each group). **m** H&E staining of TA muscles from control and *Sirt6^{mKO}* mice after repeated injury (n=4 for each group). Scale bar: 20 µm. **n** Glucose tolerance test (GTT) of control (n=4) and *Sirt6^{mKO}* mice (n=3). Two-way ANOVA test with Bonferroni multiple comparisons test: ns: p>0.05. **o** Expression of selected glycolytic genes in freshly isolated MuSCs from control and *Sirt6^{mKO}* mice. Results were obtained by RNAseq and are presented as RPKM (Reads per kilobase of transcript per Million mapped reads) (Unpaired two-tailed t-test: *p=0.0139, n=3). **p** RT-qPCR expression analysis of glycolytic genes in muscles from control and *Sirt6^{mKO}* mice. *m36b4* was used as a reference (Unpaired two-tailed t-test: p>0.05, **p =0.0048; n=3 for each group). 12-weeks old male mice were used. Data are presented as mean ± SEM. Source data are provided in the Source Data file.

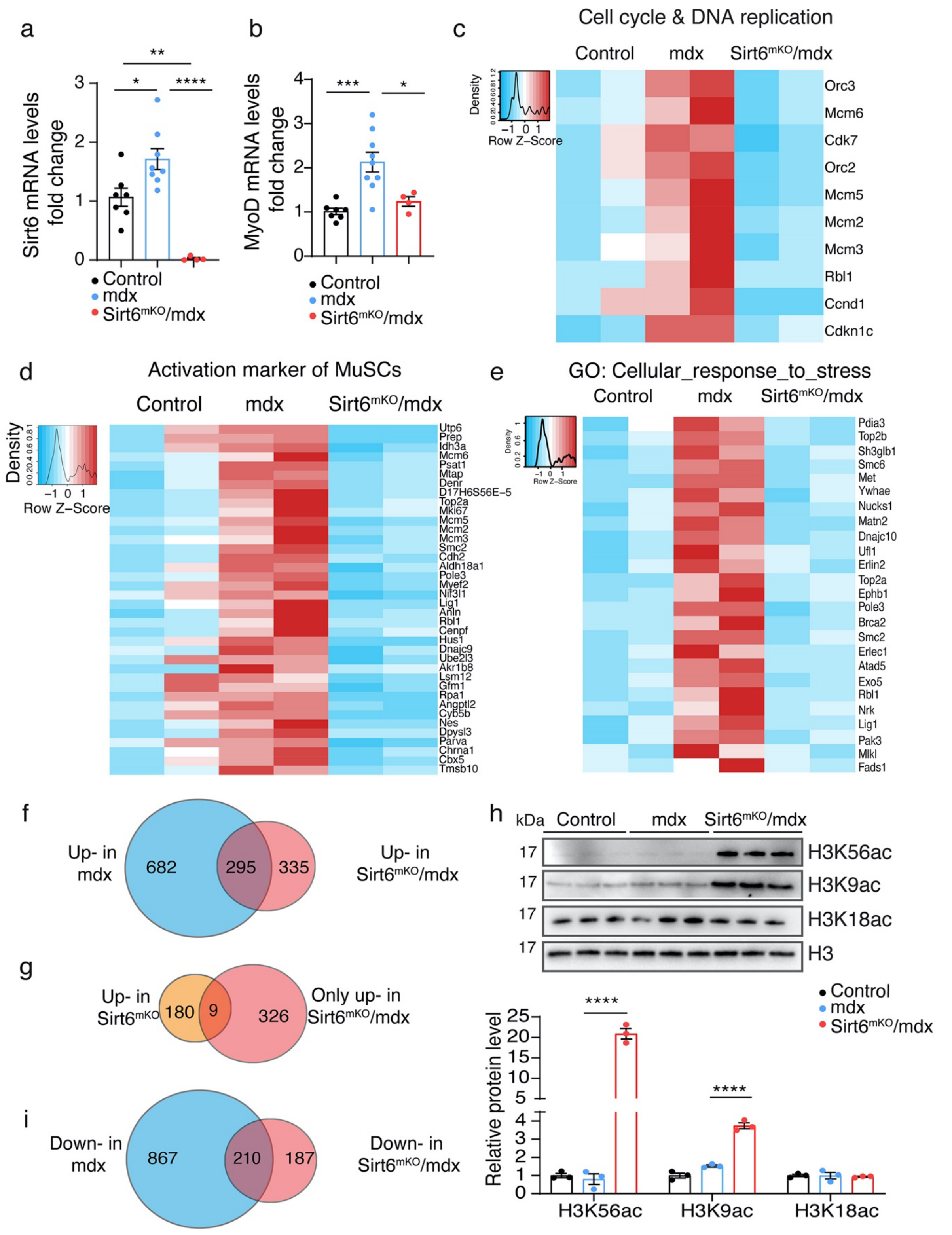

Supplementary Figure 3. Loss of *Sirt6* attenuates persistent activation of MuSCs in mdx muscles. a RT-qPCR analysis of *Sirt6* expression in MuSCs isolated from control (n=7), *mdx* (n=8) and *Sirt6^{mKO}/mdx* (n=4) mice. *m36b4* was used as a reference gene (One-way ANOVA test with Bonferroni multiple comparisons test: *p=0.024, **p=0029, ****p<0.0001.) **b** RT-qPCR analysis of *Myod* expression in freshly isolated MuSCs from control (n=7), *mdx* (n=9) and *Sirt6^{mKO}/mdx* (n=4) mice. *m36b4* was used as a reference gene (One-way ANOVA test with Bonferroni multiple comparisons test: *p=0.0197, ***p<0.0007). **c-e** Heat map of genes associated with cell cycle (c), activation markers of MuSCs (d) and cellular stress response (e)

in control, *mdx* and *Sirt6*^{mKO}*/mdx* MuSCs that were up-regulated in *mdx* MuSCs but normalized to control level in *Sirt6*^{mKO}*/mdx* MuSCs (n=2). **f** Venn diagram to identify the overlap of upregulated genes in *mdx* MuSCs and in *Sirt6*^{mKO}*/mdx* compared to control MuSCs. **g** Venn diagram to identify the overlap of upregulated genes in *Sirt6*^{mKO} MuSCs and genes that are only upregulated in *Sirt6*^{mKO}*/mdx* but not in mdx MuSCs. **h** Western blot analysis of H3K56ac, H3K9ac, and H3K18ac levels in freshly isolated MuSCs from control, *mdx* and *Sirt6*^{mKO}*/mdx* mice. Histone H3 was used as a loading control. Quantification of protein bands is shown is in the lower panel (One-way ANOVA test with Bonferroni multiple comparisons test: ****$p < 0.0001$; n=3 for each group). **i** Venn diagram to identify the overlap of downregulated genes in *mdx* MuSCs and in *Sirt6*^{mKO}*/mdx* MuSCs compared to control MuSCs. 12-weeks old male mice were used. Data are presented as mean ± SEM (a, b, h). Source data are provided in the Source Data file.

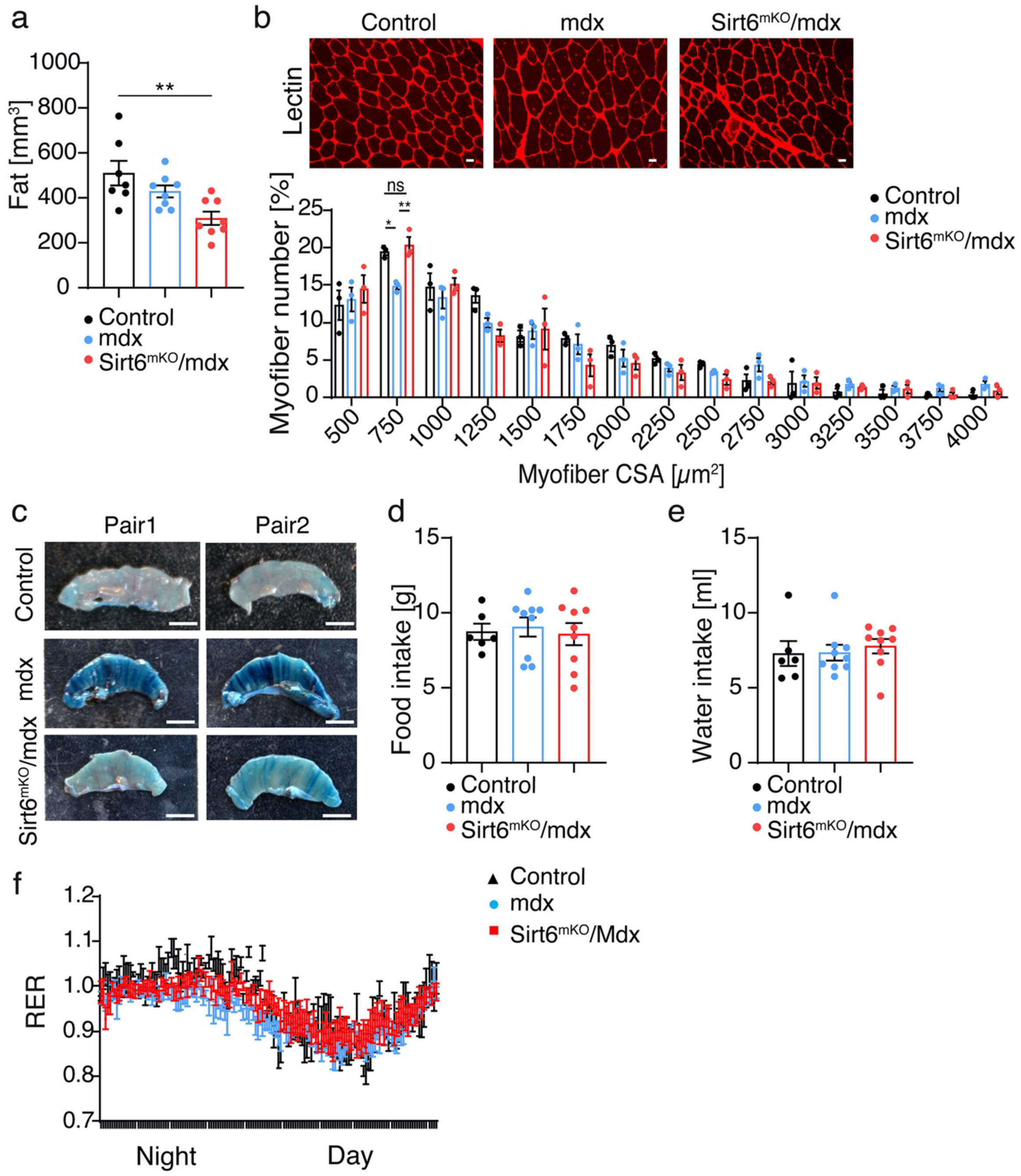

Supplementary Figure 4. Inactivation of *Sirt6* improves functions of skeletal muscles in mdx mice without apparent changes in muscle metabolism. a MRI of fat mass in 12-week-old control (n=7), *mdx* (n=8) and *Sirt6*^{mKO}*/mdx* (n=8) littermates. Quantifications of muscle and fat volume are shown in the right panel (One-way ANOVA test with Bonferroni multiple comparisons test: **p=0.0038;). **b** Lectin staining of TA muscle sections from control, *mdx* and *Sirt6*^{mKO}*/mdx* mice. Quantification of CSA of myofibers is shown in the right panel (Two-way ANOVA with Benjamini and Yekutieli multiple comparisons test: ns p>0.05, *p=0.0197, **p=0.0053; n=3 for each group). Scale bar: 20 µm. **c** Evans blue staining of muscles from control, *mdx* and *Sirt6*^{mKO}*/mdx* mice. The macroscopic view displays diaphragm muscles 16 hours after 1% Evans blue injection. Scale bar: 5 mm. **d, e** Food intake (d) and water intake (e) of control (n=6), *mdx* (n=9) and *Sirt6*^{mKO}*/mdx* (n=9) mice after 48 h acclimatization in PhenoMaster metabolic cages (One-way ANOVA test with Bonferroni multiple comparisons test: ns p>0.05). **f** Respiratory exchange ratio (RER) of control (n=3), *mdx* (n=7) and *Sirt6*^{mKO}*/mdx* (n=7) mice after 48 h acclimatization in PhenoMaster analysis metabolic cages.

12-16-weeks old male mice were used. Data are presented as mean ± SEM (a-b, d-f). Source data are provided as a Source Data file.

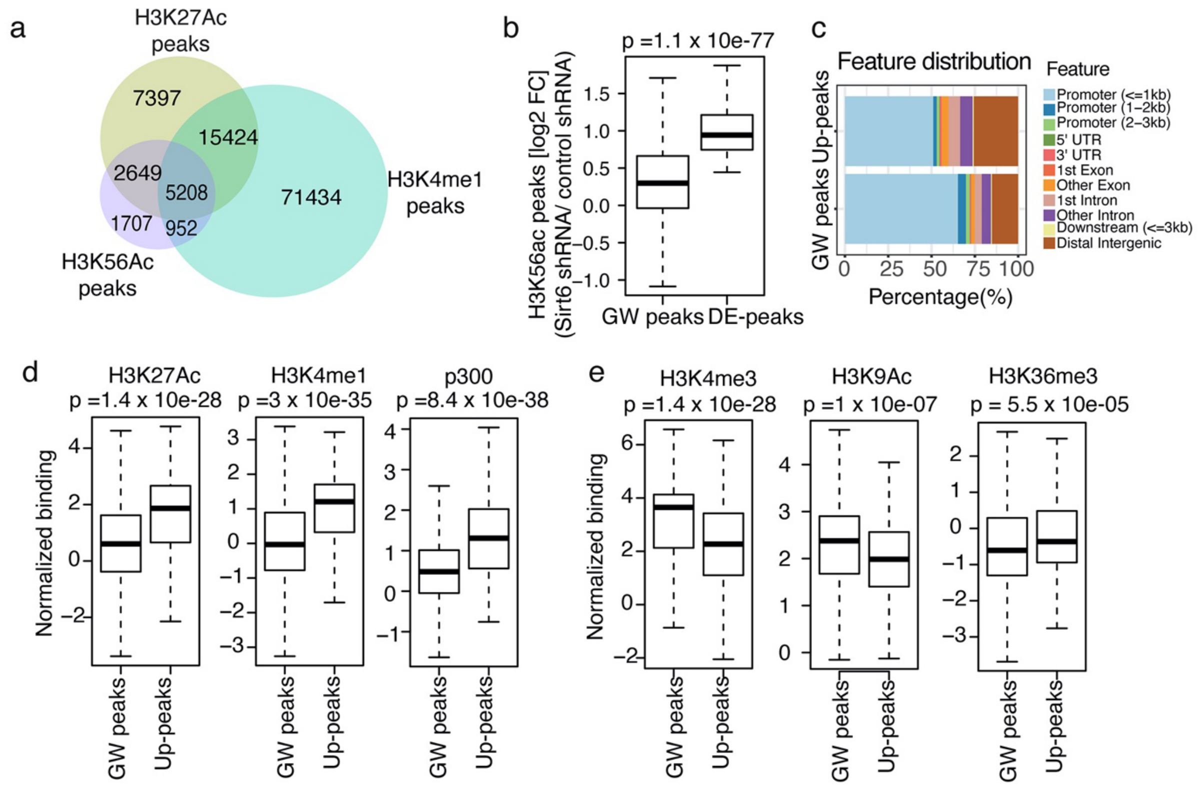

Supplementary Figure 5. SIRT6 regulates H3K56ac levels in enhancer regions of ESCs. a Venn diagram of H3K56ac, H3K27ac (ENCODE), H3K4me1 (ENCODE) ChIP-seq data in mESCs showing that H3K56ac peaks in mESCs overlap with enhancer marks (H3K27ac and H3K4me1). **b** Box plot of log2-fold change (FC) representing all H3K56ac genome wide (GM) peaks and significantly deregulated (DE) H3K56ac peaks in Sirt6^{KD} mESCs. **c** Distribution of H3K56ac ChIP-seq peaks across genomic regions. **d** Box plots indicating that H3K56ac sites induced in Sirt6^{KD} mESCs correlate stronger with enhancer marks (H3K27ac, H3K4me1, and p300 (ENCODE data sets) than all H3K56ac peaks (Wilcoxon rank sum test, $p<2.2x10^{-16}$). **e** Box plots demonstrating that H3K56ac sites induced in Sirt6^{KD} mESCs are not correlated with promoter-associated marks such as H3K4me3 and H3K9ac and only mildly enriched at gene bodies marked by H3K36me3 (ENCODE data sets). Box plots in (b, d and e) were defined by default in R, 1.5×IQR (interquartile range). The whiskers show the minimum and maximum values. Wilcoxon rank sum test, two-sided p<2.2x1016 (n=2).

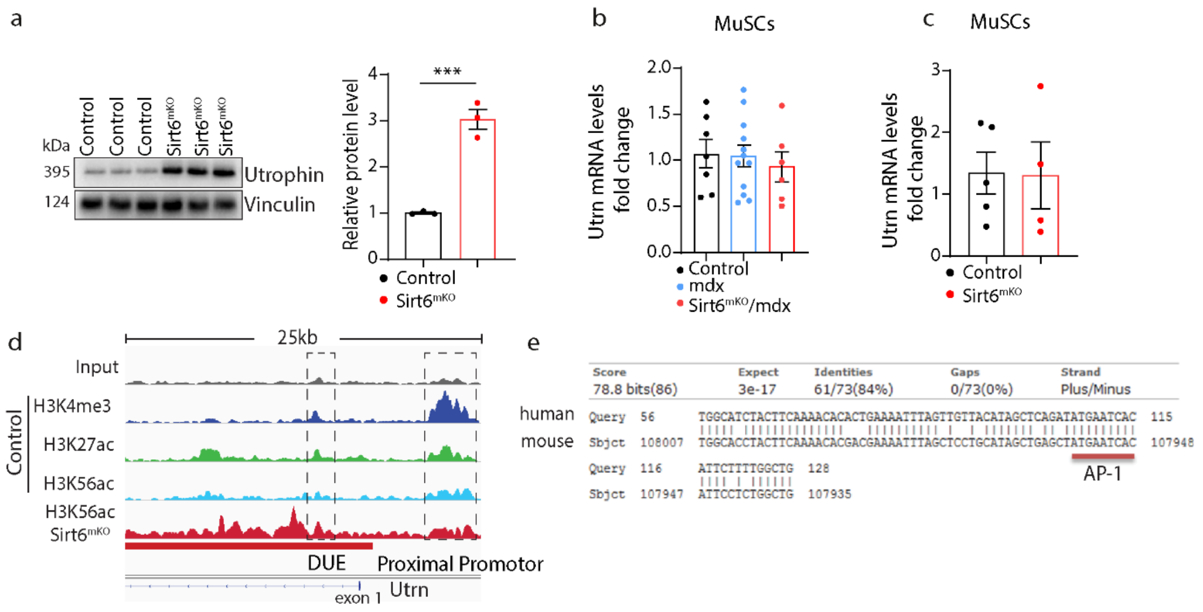

Supplementary Figure 6. SIRT6 represses activity of the DUE regulating *Utrn* gene expression. a Western blot analysis of utrophin expression in muscle tissues from control and *Sirt6*^{mKO} mice. Vinculin was used as loading control. Quantification of protein bands are shown on the right (Unpaired two-tailed t-test: ***p=0.007, n=3 for each group) **b** RT-qPCR analysis of *Utrn* gene expression in freshly isolated MuSCs from control (n=7), *mdx* (n=12) and *Sirt6*^{mKO}*/mdx* (n=6) mice. *m36b4* was used as a reference gene. (One-way ANOVA test with Bonferroni multiple comparisons test: ns p>0.05) **c** RT-qPCR analysis of Utrn gene expression in freshly isolated control (n=5) and *Sirt6*^{mKO} (n=4) MuSCs. *m36b4* was used as reference gene (Unpaired two-tailed t-test: ns p>0.05). **d** Distribution of H3K56ac, H3K4me3 and H3K27ac in the proximal promoter region and DUE of the *Utrn* gene, revealing co-localization of increased H3K56ac peaks in *Sirt6*^{mKO} MuSCs with the enhancer mark H3K27ac. **e** BLASTN sequence alignment of DUEs in human (upper) and mouse (lower) genomes. The putative AP-1 (red line) binding site is 100% conserved. 12-16-weeks old male mice were used. Data are presented as mean ± SEM (a-c). Source data are provided in the Source Data file.

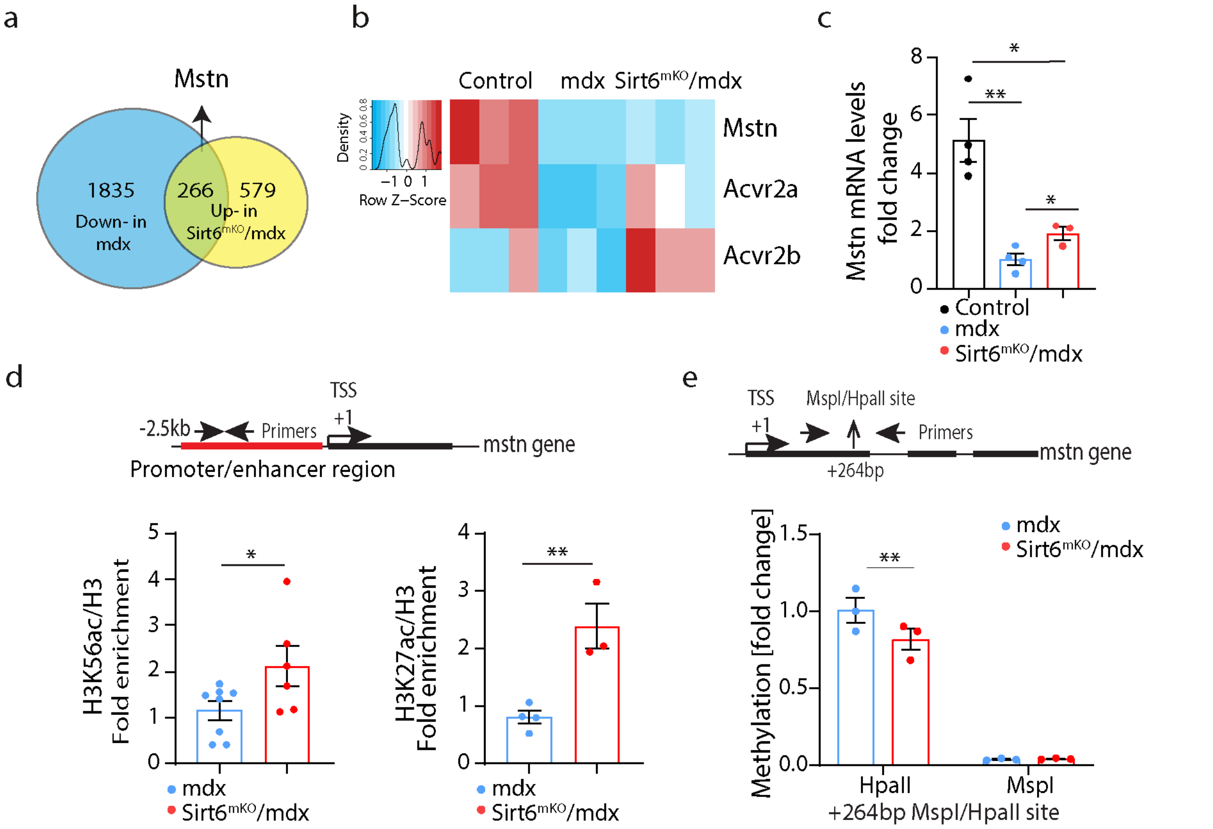

Supplementary Figure 7. Inactivation of *Sirt6* in mdx mice results in increased *Mstn* expression in skeletal muscles. a Venn diagram of overlapping downregulated genes in *mdx* compared to control muscle and upregulated genes in *Sirt6^{mKO}/mdx* compared to *mdx* muscle based in RNA-seq. **b** Heat map showing normalization of *Mstn* and its receptors in *Sirt6^{mKO}/mdx* back to wild-type level when compared to *mdx* muscles (n=3) based on DESeq normalized counts. **c** RT-qPCR analysis of *Mstn* expression in muscles from control (n=4), *mdx* (n=4) and *Sirt6^{mKO}/mdx* (n=3) mice (Unpaired two-tailed t-test: *p=0.0310, *p=0.0157, **p=0.0017). *m36b4* was used as reference gene. **d** ChIP-qPCR analysis of H3K56ac in *mdx* (n=8) and *Sirt6^{mKO}/mdx* (n=6) muscles and H3K27ac at enhancer/promoter regions of the *Mstn* gene in mdx (n=4) and *Sirt6^{mKO}/mdx* (n=3) muscles. Enrichment of H3K56ac and H3K27ac was normalized to H3. (Unpaired two-tailed t-test: *p=0.0474, **p=0.0062). **e** Methylation-sensitive PCR to detect DNA methylation at the *Mstn* promoter in muscles of *mdx* and *Sirt6^{mKO}/mdx* (Two-way ANOVA test with Bonferroni multiple comparisons test: ** p=0.0029; n=3). 12-16-weeks old male mice were used. Data are presented as mean ± SEM (c-e). Source data are provided in the Source Data file.

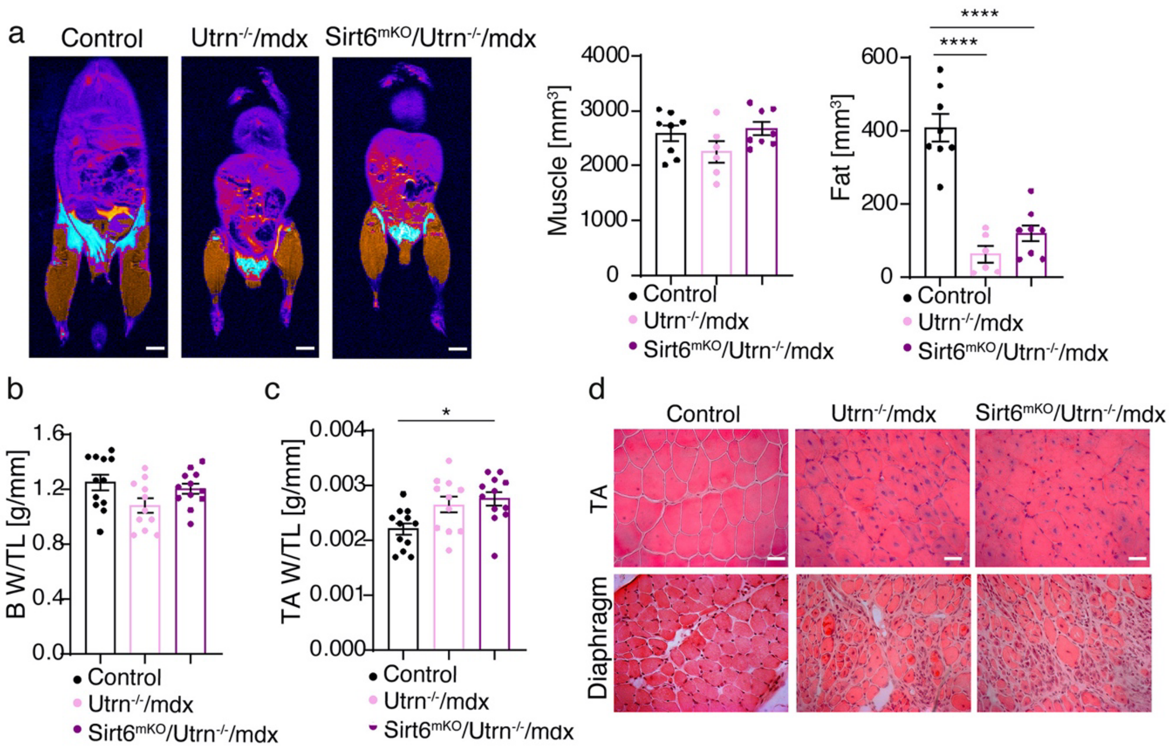

Supplementary Figure 8. Deletion of *Utrn* abrogates beneficial effects of *Sirt6* inactivation in *Sirt6^{mKO}/mdx* mice. a MRI analysis of muscle and fat mass of control (n=8), *Utrn$^{-/-}$/mdx* (n=6) and *Sirt6^{mKO}/Utrn$^{-/-}$/mdx* (n=8) littermates. Scale bar: 5 mm. Quantification of muscle and fat volume is shown on the right. (One-way ANOVA with Bonferroni multiple comparisons test: ****p<0.0001). **b** TA muscle weight/tibia length ratios of control (n=12), *Utrn$^{-/-}$/mdx* (n=11) and *Sirt6^{mKO}/Utrn$^{-/-}$/mdx* (n=12) mice (One-way ANOVA with Bonferroni multiple comparisons test: *p=0.0130). **c** Body weight/tibia length ratios of control (n=12), *Utrn$^{-/-}$/mdx* (n=11) and *Sirt6^{mKO}/Utrn$^{-/-}$/mdx* (n=12) mice (One-way ANOVA with Bonferroni multiple comparisons test: ns p>0.05). **d** H&E staining of TA (upper panel) and diaphragm muscles (lower panel) in control (n=3), *Utrn$^{-/-}$/mdx* (n=3) and *Sirt6^{mKO}/Utrn$^{-/-}$/mdx* (n=3) mice. Scale bar: 20 µm. 5-8-weeks old male and female mice were used. Data are presented as mean ± SEM. Source data are provided in the Source Data file.

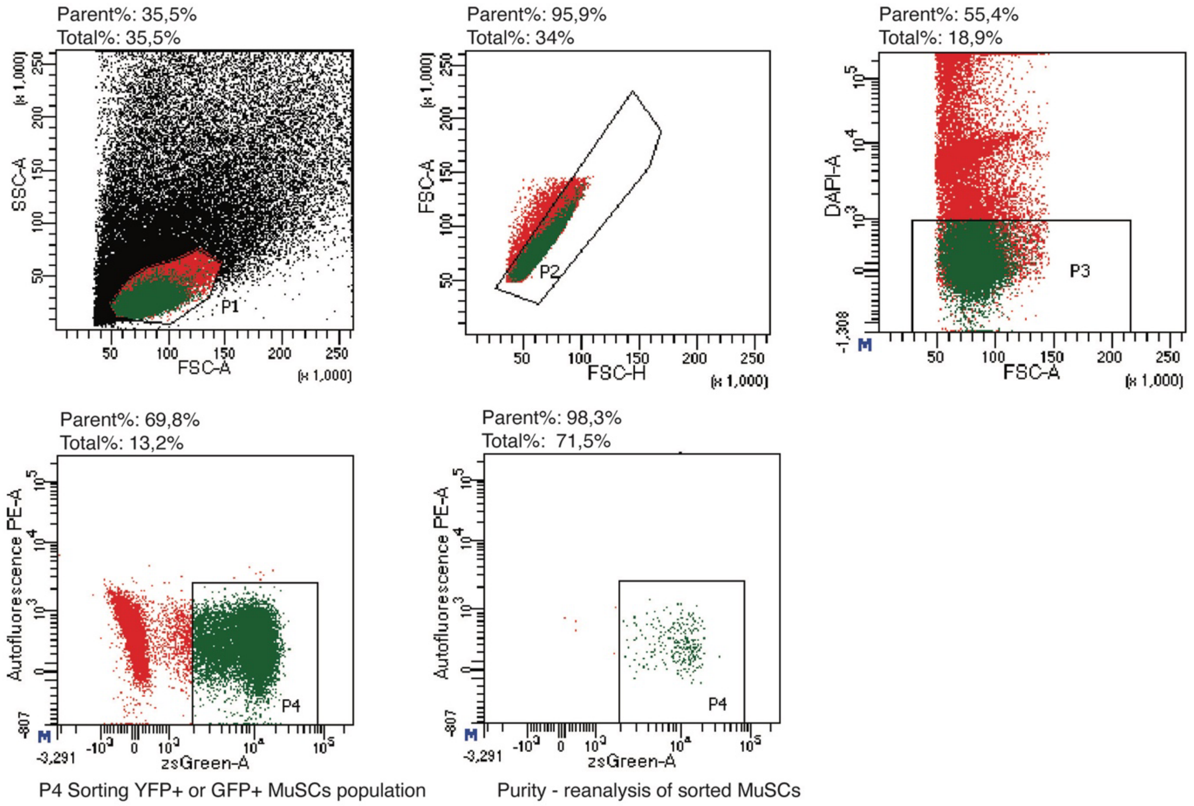

Supplementary Figure 9. Detailed cell sorting strategy for Integrin-a7+/CD34+ MuSCs population using CD FACS Diva v8 software (parent and total percentage is shown in individual figures).

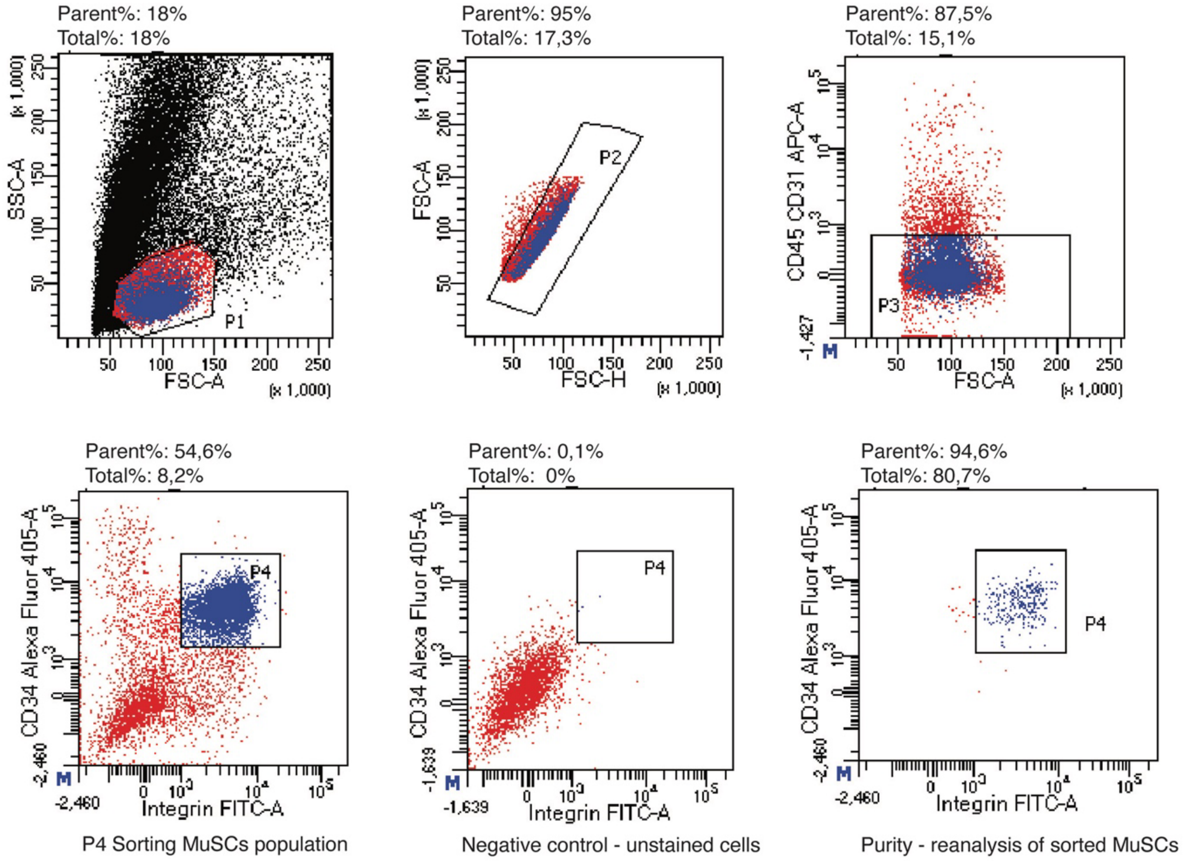

Supplementary Figure 10. Detailed cell sorting strategy for YFP+ or GFP + MuSCs population using CD FACS Diva v8 software (parent and total percentage is shown in individual figures).

Supplementary Table 1. List of gene names displayed in the heat map of Supplementary Figure 1c

Uhrf1	Ccng1	Lmnb1	Smc2	Smc4	Cdkn2a
Eef1e1	Smc6	Anxa1	Erp44	Parpbp	Myof
Axl	Ywhae	Capn1	Tnc	Fignl1	Lig4
Ralb	Top2a	Smc5	Pole3	Melk	Tmco1
Cav1	Snw1	Prdx3	Txndc12	Rps27l	Nrk
Mgst1	Tmx1	Sfr1	Exo5	Sh3glb1	Pla2r1
Met	Mnat1	Casp12	Spp1	Cdkn3	Nod2
Fads1	Thbs4	Cops5	Rad51ap1	Dap	Lig1
Mlkl	Psmc6	Nucks1	Pak1	Neil3	Zfp110
Pten	Rnase4	Prrx1	Bccip	Cdk6	Serpinb6a
Ehmt2	Rnaseh2b	Psmd14	Pak3	Slc25a24	Ufc1
Etaa1	Dnajc15	Dnajc10	Erlin2	Nsmce4a	Zbtb18
Top2b	Pbk	Creb3l1	Casp3	Ufl1	Ifi204
Atad5	Matn2	Pdia3	Tipin	Brca2	Srpx
Cdk1	Stk3	Rbl1	Arpp21	Mdfic	
Actr2	Snai2	Ect2	Ephb1	Phlda3	
Erlec1	Chaf1b	Ufm1	Gtf2h5	Wipi1	

Supplementary table 2. List of gene names displayed in the heat map of Figure 4e

Axl	Xbp1	Ptpn22	Casp4	Rap2a	Scn2a
Prkd1	Rnase4	Bag3	Pdk3	Cfl1	Hspa1b
Hspa8	Dab2	Cryab	Macrod1	Flot1	Hspa1a
Cd34	Shisa5	Usp28	Cdk6	Hspa2	
Lamp2	Lcn2	Arpp21	Cdkn2a	Ifi204	

Supplementary table 3. List of gRNAs used to target mouse DUE

gRNA ID	sgRNA targeting sequence (5'->3')	Ensemble gene ID
mDUEgRNA#6	TCCCTTTGAATGGACCCTAC	chr10:12858221-12858240
mDUEgRNA#7	GCATAGCTGAGCTATGAATC	chr10:12858272-12858291
mDUEgRNA#8	AACTGTGCCCTCCCTTTGAA	chr10:12858214-12858233
mDUEgRNA#9	CTGGATTGTGTTGCAAAAGC	chr10:12858304-12858323
mDUEgRNA#10	CTGGCTGGATTGTGTTGCAA	chr10:12858300-12858319
mDUEgRNA#11	AGCTATGAATCACATTCCTC	chr10:12858281-12858300
Non-targeting gRNA	GCGCGAATCGCGATATACCG	

Supplementary table 4. List of antibodies used in this study and their applications

Antibody	Application (dilution)	Manufacturer	Cat. No.
**Anti-Pax7 mouse**	IF (1:1000)	R&D Systems	MAB1675
**Anti-MyoD rabbit**	IF (1:1000)	Santa Cruz	SC-304
**Anti-Gapdh**	WB (1:2000)	Cell signaling	(14C10)
**Rabbit IgG**	ChIP	Diagenode	C15410206
**Anti-Sca1 APC**	FACS (1:100)	eBioscience	17-5981-83
**Anti-CD45 APC**	FACS (1:100)	eBioscience	17-0451-83
**Anti-CD31 APC**	FACS (1:100)	eBioscience	17-0311-82
**Anti-APC MicroBeads**		MACS	130-090-855
**Integrin-a7 FITC**	FACS (1:100)	MBL,JP	K0046-4
**Anti-CD34 A450**	FACS (1:100)	eBioscience	48-0341-82
**H3**	WB (1:2000), ChIP	Cell Signaling	9715L
**H3K56ac**	WB (1:1000), ChIP	EpiGentek	A-4026-050-EP
**H3K9ac**	WB (1:1000)	Abcam	ab10812
**H3K18ac**	WB (1:1000)	Cell signaling	#9675
**H3K27ac**	ChIP	Abcam	ab8895
**SirT6 (D8D12)**	WB (1:1000)	Cell signaling	#12486
**P300**	ChIP	Active motif	61903
**cJun (60A8)**	ChIP	Cell signaling	9165
**H3K56ac**	WB, ChIP	Cell signaling	4243S
**Utrophin**	WB (1:100)	Santa Cruz's	sc-33700
**Anti-HA tag antibody**	ChIP	Abcam	ab9110

IF: Immunofluorescence, WB: Western blot, ChIP: Chromatin immunoprecipitation

Supplementary table 5. A characterization of immortalized human myoblast cell lines

Sample	Gender	Year old
Control 1	male	13-38 years old
Control 2	male	
Control 3	male	
Control 4	female	
DMD 1	male	13-16 years old
DMD 2	male	
DMD 3	male	
DMD 4	male	
