# Supplementary material for: Inactivation of Sirt6 ameliorates muscular dystrophy in mdx mice by releasing suppression of utrophin expression
Source: Nat Commun. 2022 Jul 20;13:4184. doi: 10.1038/s41467-022-31798-z (PMC9300598; doi:10.1038/s41467-022-31798-z)
Supplement: Supplementary file 3 — Description of Additional Supplementary Files [file 41467_2022_31798_MOESM3_ESM.pdf]

Description of Additional Supplementary Files

File Name: Supplementary Data 1
Description: List of primers used for genotyping, RT-qPCR and chromatin IP.
