# Supplementary material for: Inactivation of Sirt6 ameliorates muscular dystrophy in mdx mice by releasing suppression of utrophin expression
Source: Nat Commun. 2022 Jul 20;13:4184. doi: 10.1038/s41467-022-31798-z (PMC9300598; doi:10.1038/s41467-022-31798-z)
Supplement: Supplementary file 5 — Reporting Summary [file 41467_2022_31798_MOESM5_ESM.pdf]

nature research

Corresponding author(s): Thomas Braun; Xuejun Yuan

Last updated by author(s): Jun 14, 2021

Reporting Summary

Nature Research wishes to improve the reproducibility of the work that we publish. This form provides structure for consistency and transparency in reporting. For further information on Nature Research policies, see our Editorial Policies and the Editorial Policy Checklist.

Statistics

For all statistical analyses, confirm that the following items are present in the figure legend, table legend, main text, or Methods section.

n/a	Confirmed	
☐	☒	The exact sample size (*n*) for each experimental group/condition, given as a discrete number and unit of measurement
☐	☒	A statement on whether measurements were taken from distinct samples or whether the same sample was measured repeatedly
☐	☒	The statistical test(s) used AND whether they are one- or two-sided<br>*Only common tests should be described solely by name; describe more complex techniques in the Methods section.*
☒	☐	A description of all covariates tested
☐	☒	A description of any assumptions or corrections, such as tests of normality and adjustment for multiple comparisons
☐	☒	A full description of the statistical parameters including central tendency (e.g. means) or other basic estimates (e.g. regression coefficient) AND variation (e.g. standard deviation) or associated estimates of uncertainty (e.g. confidence intervals)
☐	☒	For null hypothesis testing, the test statistic (e.g. *F*, *t*, *r*) with confidence intervals, effect sizes, degrees of freedom and *P* value noted<br>*Give P values as exact values whenever suitable.*
☒	☐	For Bayesian analysis, information on the choice of priors and Markov chain Monte Carlo settings
☒	☐	For hierarchical and complex designs, identification of the appropriate level for tests and full reporting of outcomes
☒	☐	Estimates of effect sizes (e.g. Cohen's *d*, Pearson's *r*), indicating how they were calculated

Our web collection on statistics for biologists contains articles on many of the points above.

Software and code

Policy information about availability of computer code

Data collection

Zeiss Z1.Imager - software Zen2 6.1.7601
Leica Sp8 - Software LAS X 3.5.7.23225
FACS AriaTM III (BD Biosciences) - BD FACS Diva v8 Software

Data analysis

GraphPad PRISM 8
StepOne Software v2.3
ImageJ 1.53
R 3.4
R 3.11
DESeq2 1.16.1
DESeq2 version 1.62
Macs2 2.1.0
MACS2 2.1.1
STAR 2.4.0a
GenomicRanges 1.28.6
IGV version 2.4.10
trim_galore 0.4.1

RNA-seq analysis
Library quality was controlled uisng a Bioanalyzer 2100 and final sequencing was performed with Ion PI Sequencing 200 Kit v2 (Thermo Fisher). Raw reads were assessed for quality, adaptor content and duplication rates with FastQC 0.10.1, trimmed by Reaper version 13–100 and terminally aligned to the Ensemble mouse genome version mm10 (GRCm38) by STAR 2.4.0a. The number of reads aligning to genes was

counted with featureCounts 1.4.5-p1 tool from the Subread package. Only reads mapping at least partially inside exons were admitted and aggregated per gene. Reads overlapping multiple genes or aligning to multiple regions were excluded. Differentially expressed genes were identified using DESeq2 version 1.62 l. Only genes with a minimum fold change of +- 2, a maximum Benjamini-Hochberg corrected p-value of 0.05, and a minimum combined mean of 5 reads were assumed to be significantly differentially expressed. The Ensemble annotation was enriched with UniProt data (release 06.06.2014) based on Ensembl gene identifiers (Activities at the Universal Protein Resource (UniProt)). Correlations of replicate gene counts were assessed with the Spearman ranked correlation algorithm included in R 3.11 (R: A language and environment for statistical computing). Volcano plots were computed using the script run_DE_analysis.pl included in Trinity version 20140717, which employs R functions for plotting. Further global clustering of samples was performed using the regularized-logarithm transformation method of DESeq2, based on complete euclidean distances and hierarchical clustering.

ChIP-seq analysis
Sequencing was performed with the NextSeq500 instrument (Illumina) using v2 chemistry with 1x75bp single end setup.
FASTQ files were controlled for quality issues, using FastQC (https://www.bioinformatics.babraham.ac.uk/projects/fastqc/). Quality aware trimming of reads and adapter removal were performed using Trim Galore (https://www.bioinformatics.babraham.ac.uk/projects/trim_galore/). Read alignment against the mm10 mouse genome was done against a reference downloaded as a pre-compiled BWT index from Illumina's iGenome repository (https://emea.support.illumina.com/sequencing/, sequencing_software/igenome.html). Read alignment was performed by using bowtie version 1.1.2 with parameters -k 1 -m 1. Duplicate removal was performed by using Picard's MarkDuplicates function. Coverage vectors were generated with Deeptools bamCoverage function, using RPKM (reads per kilo base per million mapped reads) normalization. Visualization of binding profiles was done by using the R/BioConductor package Gviz or the Integrative Genome Viewer (IGV). Peak calling was done using MACS2. The resulting set was filtered against blacklisted chromatin regions, as detected by ENCODE. Read counts across peaks were determined using the featureCounts function of the Subread package. Differential binding analysis was performed by using DESeq2 after merging overlapping peaks into reference peak sets, using the reduce function of the BioConductor GenomicRanges package. H3K56ac data were compared to available data for histone modifications and other chromatin modifiers in mES cells from ENCODE by downloading the raw sequencing reads of the first replicate for H3K27ac, H3k4me1, H3K4me3, H3K9ac, H3K36me3 and p300 through the UCSC browser data download portal (https://hgdownload-test.gi.ucsc.edu/goldenPath/mm9/encodeDCC/wgEncodeLicrTfbs/).

ATAC-seq analysis
Libraries were mixed in equimolar ratios and sequenced on the NextSeq500 platform using V2 chemistry with paired-end mode. Quality control of FASTQ files was done using FastQC. Quality aware trimming of reads and adapter removal were performed using Trim Galore. Reads were aligned to a pre-compiled index of the mouse mm10 genome using BWA with default settings. Duplicate reads were removed with Samtools' rmdup function. Virtual footprinting for motif analysis was done by adapting the HINT subroutine of the Regulatory Genomics Toolbox suite (http://www.regulatory-genomics.org/). Peaks were called by using MACS2 (version 2.1.1.20160309) with the default setting of the p-value threshold to -p 0.01 and using the -bampe option. Coverage bigwig files were generated with Deeptools bamCoverage function at 50 bp resolution.

For manuscripts utilizing custom algorithms or software that are central to the research but not yet described in published literature, software must be made available to editors and reviewers. We strongly encourage code deposition in a community repository (e.g. GitHub). See the Nature Research guidelines for submitting code & software for further information.

Data

Policy information about availability of data

All manuscripts must include a data availability statement. This statement should provide the following information, where applicable:

- Accession codes, unique identifiers, or web links for publicly available datasets
- A list of figures that have associated raw data
- A description of any restrictions on data availability

GEO accession number: GSE168330 Figure Supplementary Figure 5a, 5b, 5c and 5d are associated with this data.
GEO accession number: GSE168331 Figure 5a, 5b, 5c, 5d and 5e are associated with this data.
GEO accession number: GSE168329 Figure 5f is associated with this data.
GEO accession number: GSE168983 Figure 1a, 1b, Figure 2c, Supplementary Figure 1c, Supplementary Figure 3c, 3d, 3e, 3f and 3i are associated with this data.
GEO accession number: GSE168984 Figure 4a, 4b, 4c, 4d, 4e and Supplementary Figure 7a and 7b are associated with this data.
GEO accession number: GSE199487 Supplementary Figure 2c, 2d, 2e and Supplementary Figure 3g.
GEO accession number: GSE103163 Figure 5a and 5e are associated with this data.

Field-specific reporting

Please select the one below that is the best fit for your research. If you are not sure, read the appropriate sections before making your selection.

☒ Life sciences ☐ Behavioural & social sciences ☐ Ecological, evolutionary & environmental sciences

For a reference copy of the document with all sections, see nature.com/documents/nr-reporting-summary-flat.pdf

Life sciences study design

All studies must disclose on these points even when the disclosure is negative.

Sample size

Sample size were determined based on established practice and applicable standards. We opted for sample sizes which are commonly used sample sizes in the field.
For in vivo studies, a minimum of three biological replicates was analyzed. Each experiment in which data were quantified was performed with at least 3 replicates.

Data exclusions	No data were excluded.
Replication	All in vivo studies were performed once with indicated numbers of animals. Sample sizes and statistical analyses and significance levels are all indicated in the figure legends or the method part.
Randomization	All animals were numbered and experiments were performed in a blinded pattern. After data collection, genotypes were revealed and animals assigned to different groups for analysis.
Blinding	In vivo experiments were performed in a blinded pattern.<br>In vitro experiments were not blinded during data collection or analysis. Positive controls, negative controls and target samples were analyzed in exactly the same manner.

Reporting for specific materials, systems and methods

We require information from authors about some types of materials, experimental systems and methods used in many studies. Here, indicate whether each material, system or method listed is relevant to your study. If you are not sure if a list item applies to your research, read the appropriate section before selecting a response.

Materials & experimental systems

n/a	Involved in the study	
☐	☒	Antibodies
☐	☒	Eukaryotic cell lines
☒	☐	Palaeontology and archaeology
☐	☒	Animals and other organisms
☒	☐	Human research participants
☒	☐	Clinical data
☒	☐	Dual use research of concern

Methods

n/a	Involved in the study	
☐	☒	ChIP-seq
☐	☒	Flow cytometry
☒	☐	MRI-based neuroimaging

Antibodies

Antibodies used	Anti-Pax7 mouse IF (1:1000) R&D Systems MAB1675<br>Anti-MyoD rabbit IF (1:1000) Santa Cruz SC-304<br>Anti-Gapdh WB (1:2000) Cell signaling (14C10)<br>Rabbit IgG ChIP Diagenode C15410206<br>Anti-Sca1 APC FACS (1:100) eBioscience 17-5981-83<br>Anti-CD45 APC FACS (1:100) eBioscience 17-0451-83<br>Anti-CD31 APC FACS (1:100) eBioscience 17-0311-82<br>Integrin-a7 FITC FACS (1:100) MBL,JP K0046-4<br>Anti-CD34 A450 FACS (1:100) eBioscience 48-0341-82<br>H3 WB (1:2000), ChIP Cell Signaling 9715L<br>H3K56ac WB (1:1000), ChIP EpiGentek A-4026-050-EP<br>H3K9ac WB (1:1000) Abcam ab10812<br>H3K18ac WB (1:1000) Cell signaling #9675<br>H3K27ac ChIP Abcam ab8895<br>SirT6 (D8D12) WB(1:1000) Cell signaling #12486<br>P300 ChIP Active motif 61903<br>c-Jun (60A8) ChIP Cell signaling 9165<br>H3K56ac WB, ChIP Cell signaling 4243S<br>Utrophin WB (1:100) Santa Cruz's sc-33700<br>Anti-HA tag antibody ChIP Abcam ab9110
Validation	All antibodies used in this study were validated by the manufactureres or by ourselves using material from genetic knockout models when available or by using only secondary without primary antibodies.<br>Integrin-a7 FITC for FACS was validated by comparing Integrin-a7 FITC staining with expression of a tomato reporter gene, specifically active in muscle stem cells.<br><br>Pax7 (1:1000 R&D Systems MAB1675) validated by immunocytochemistry in C2C12 Mouse Cell Line; MyoD (1:1000 Santa Cruz SC-304) validated by western blot analysis in HeLa (A), HL-60 (B), SJRH30 (C), NIH/3T3 (D) and RD (E) whole cell lysates and A-673 nuclear extract (F); Gapdh (WB 1:2000 Cell signaling (14C10) validated by western blot analysis of extracts from various cell lines, immunofluorescent analysis of HeLa cells; IgG (ChIP Diagenode C15410206) validated by ChIP assays and immunofluorescence in HeLA cells; Sca1 -APC (FACS 1:100, eBioscience 17-5981-83) validated by FACS analysis of stained of unstimulated and 4-day Con A-stimulated BALB/c splenocytes; CD45-APC (FACS 1:100, eBioscience 17-0451-83) validated by FACS analysis in C57BL/6 mouse bone marrow cells; CD31 APC (FACS 1:100, eBioscience 17-0311-82) validated by FACS analysis in C57BL/6 bone marrow cells; Integrin-a7 FITC (FACS 1:100, MBL,JP K0046-4);CD34 A450 (FACS 1:100 eBioscience 48-0341-82) validated by FACS analysis and staining of C57BL/6 bone marrow cells with Anti-Mouse Hematopoietic Lineage antibodies; H3 (WB 1:2000, ChIP 2Cell Signaling 9715L)validated by western blot analysis of extracts from various cell lines; H3K56ac (WB 1:1000, ChIP EpiGentek A-4026-050-EP) validated by western blot analysis of extracts from BT-474, 293T, and H3 protein as negative control. H3K9ac (WB 1:1000 Abcam ab10812) validated by ChIP using Hela cells, by western blot analysis of extracts from Calf Thymus Histone Preparation Nuclear Lysate with

Human Histone H3 (acetyl K9) peptide; H3K18ac (WB 1:1000, Cell signaling #9675) validated by western blot analysis of extracts from NIH/3T3 cells, untreated or TSA-treated, Immunohistochemical analysis of paraffin-embedded NIH/3T3 cells, untreated (left) or TSA-treated (right), ChIP performed in HeLa cells; H3K27ac (Abcam ab8895) validated by ChIP using Hela cells, immunofluorescence in Hela cells, by western blot analysis of Untreated Mouse MEF cell lysate and 0.4 µM Trichostatin A treatment for 18 hr Mouse MEF cell lysate; SirT6 (D8D12) (Cell signaling #12486) validated by western blot analysis of extracts from various cell lines (HCT116, C2C12, H-4-II-E, COs-7), western blot analysis of extracts from HCT 116 cells, expressing either non-targeting shRNA (shNT) or shSirT6, western blot analysis of extracts from SirT6 wild-type (WT) and knockout (KO) mouse embryonic fibroblasts (MEF); p300 antibody (mAb) validated by IF on HeLa cells and ChIP-seq in LNCaP cells; c-Jun (60A8) ChIP Cell signaling 9165 validated by western blot analysis of extracts from control HeLa cells or c-Jun knockout HeLa cells; H3K56ac WB, ChIP Cell signaling 4243S validated by western blot analysis of extracts from HeLa, C6 and COS cells, untreated or treated with Trichostatin A (TSA); Utrophin WB (1:100) Santa Cruz's sc-33700 validated by western blot analysis of utrophin expression in C2C12 , Caco-2, SJRH30 and A-673 whole cell lysates; Anti-HA tag antibody ChIP Abcam ab9110 validated by western blot with a nuclear lysate of HEK293T cells transiently expressing HA-tagged protein;

Eukaryotic cell lines

Policy information about cell lines

Cell line source(s)	C2C12 source ATCC #CRL-1772<br>Human myoblast cell lines from DMD and control individuals were provided by the platform for immortalization of human cells of the Institut de Myologie (Paris, France). The initial biopsies, from which the cell lines were generated, were provided by MyoBank, the tissue bank of the Institut de Myologie in Paris, affiliated with EuroBioBank. MyoBank has received approval from the French Ministry of Higher Education, Research and Innovation to distribute human samples for research (Authorization code AC-2019-3502).
Authentication	Human cell lines were used as obtained from Institut de Myologie (Paris, France).
Mycoplasma contamination	Tested for being Mycoplasma free
Commonly misidentified lines<br>(See ICLAC register)	No commonly misidentified cell lines were used

Animals and other organisms

Policy information about studies involving animals; ARRIVE guidelines recommended for reporting animal research

Laboratory animals	The following mouse strains were used in the study:<br>Sirt6mKO were generated by crossing Sirt6flox/flox mice, generated in house, ROSA26-YFP and Pax7ICN mouse strains.<br>mdx - C57BL/10ScSn-Dmdmdx/J<br>Sirt6mKO/mdx were generated by crossing- Sirt6mKO and C57BL/10ScSn-Dmdmdx/J (mdx) mouse strains.<br>Sirt6mKO/Utrn-/-/mdx were generated by crossing Sirt6mKO/mdx and B10ScSn.Cg-Utrntm1Ked Dmdmdx/J mouse strains.<br>Pax7ZsGreen<br><br>All mice were maintained on a C57BL/6 background and littermates were used as controls in all experiments.<br><br>All in vivo experiments using control, mdx and Sirt6mKO/mdx mice were performed on male mice as muscular dystrophy is a X-linked disease affecting mainly 8-20 weeks old males.<br>All in vivo experiments using Utrn-/-/mdx and Sirt6mKO/Utrn-/-/mdx mice were performed on balanced cohorts of male and female mice since both gender exhibit severe phenotype. No phenotypic differences between 5-8 weeks old females and males were obvious.<br><br>CRISPR-dCas9 editing for in vitro experiments was accomplished using primary muscle stem cells (MuSCs) isolated from 8-20 weeks old male and female mice, since large amounts of MuSCs were required to complete the experiment.
Wild animals	Studies did not involve wild animals.
Field-collected samples	Studies did not involve samples collected in the field.
Ethics oversight	All animal experiments were done in accordance with the Guide for the Care and Use of Laboratory Animals published by the US National Institutes of Health (NIH Publication No. 85-23, revised 1996) and were approved by the responsible Committee for Animal Rights Protection of the State of Hessen (Regierungspraesidium Darmstadt, Wilhelminenstr. 1-3, 64283 Darmstadt, Germany) with the project number B2/1125, B2/2019 and B2/1137.

Note that full information on the approval of the study protocol must also be provided in the manuscript.

ChIP-seq

Data deposition

☒ Confirm that both raw and final processed data have been deposited in a public database such as GEO.

☒ Confirm that you have deposited or provided access to graph files (e.g. BED files) for the called peaks.

Data access links
May remain private before publication.

https://www.ncbi.nlm.nih.gov/geo/query/acc.cgi?acc=GSE168330
https://www.ncbi.nlm.nih.gov/geo/query/acc.cgi?acc=GSE168331

Files in database submission

GSM5136332 MuScs_Sirt6mKO rep1 H3K56ac
GSM5136333 MuScs_Sirt6mKO rep1 input DNA
GSM5136334 MuScs_Sirt6mKO rep2 H3K56ac
GSM5136335 MuScs_Sirt6mKO rep2 input DNA
GSM5136336 MuScs_Control rep1 H3K56ac
GSM5136337 MuScs_Control rep1 input DNA rep 1
GSM5136338 MuScs_Control rep2 H3K56ac
GSM5136339 MuScs_Control rep2 input DNA rep 2
GSM5136340 Scramble mESCs input DNA
GSM5136341 Scramble mESCs rep1 H3K56ac
GSM5136342 Scramble mESCs rep2 H3K56ac
GSM5136343 Sirt6KD mESCs input DNA
GSM5136344 Sirt6KD mESCs rep1 H3K56ac
GSM5136345 Sirt6KD mESCs rep2 H3K56ac

Genome browser session
(e.g. UCSC)

https://genome.ucsc.edu/s/MarekB/mm10_AG_NatCom

Methodology

Replicates

Two biological replicates for H3K56ac ChIP-seq mESC and MuSC.

Sequencing depth

GSM5136328 Control ATAC-seq rep1 atac_SC_591543_R1.fastq.gz atac_SC_591543_R2.fastq.gz 11769883 9336874 2x75bp paired-end
GSM5136329 Control ATAC-seq rep2 atac_SC_585290_R1.fastq.gz atac_SC_585290_R2.fastq.gz 17704337 11829603 2x75bp paired-end
GSM5136330 Sirt6mKO ATAC-seq rep1 atac_SC_582814_R1.fastq.gz atac_SC_582814_R2.fastq.gz 10724884 9376943 2x75bp paired-end
GSM5136331 Sirt6mKO ATAC-seq rep2 atac_SC_585291_R1.fastq.gz atac_SC_585291_R2.fastq.gz 16319524 13997101 2x75bp paired-end

GSM5136332 MuScs_Sirt6mKO rep1 H3K56ac sKO_1_H3K56_R1_trimmed.fastq.gz 36180821 29167706 1x64 bp single-end
GSM5136333 MuScs_Sirt6mKO rep1 input DNA sKO_1_R1_trimmed.fastq.gz 34275128 29699423 1x64 bp single-end
GSM5136334 MuScs_Sirt6mKO rep2 H3K56ac sKO_2_H3K56_R1_trimmed.fastq.gz 31532670 29167706 1x64 bp single-end
GSM5136335 MuScs_Sirt6mKO rep2 input DNA sKO_2_R1_trimmed.fastq.gz 37250193 33715962 1x64 bp single-end
GSM5136336 MuScs_Control rep1 H3K56ac WT1_h3k56ac_R1_trimmed.fastq.gz 36514802 28735388 1x64 bp single-end
GSM5136337 MuScs_Control rep1 input DNA rep 1 WT1_input_R1_trimmed.fastq.gz 48341713 40375700 1x64 bp single-end
GSM5136338 MuScs_Control rep2 H3K56ac WT2_h3k56ac_R1_trimmed.fastq.gz 34665232 24966661 1x64 bp single-end
GSM5136339 MuScs_Control rep2 input DNA rep 2 WT2_input_R1_trimmed.fastq.gz 34679473 28981672 1x64 bp single-end

GSM5136340 Scramble mESCs input DNA ctrl1_trim.fastq.gz 17289627 14369001 1x80bp single-end
GSM5136341 Scramble mESCs rep1 H3K56ac h3k56ac-1_trim.fastq.gz 23937281 20743369 1x80bp single-end
GSM5136342 Scramble mESCs rep2 H3K56ac h3k56ac-2_trim.fastq.gz 18035437 15877466 1x80bp single-end
GSM5136343 Sirt6KD mESCs input DNA sirt6_input_1_trim.fastq.gz 27374690 25557021 1x80bp single-end
GSM5136344 Sirt6KD mESCs rep1 H3K56ac sirt6_k56_1_trim.fastq.gz 21066098 19680012 1x80bp single-end
GSM5136345 Sirt6KD mESCs rep2 H3K56ac sirt6_k56_2_trim.fastq.gz 22499289 20900589 1x80bp single-end

Antibodies

H3K56ac, EpiGentek, A-4026-050-EP

Peak calling parameters

Peak calling was done using MACS2 using default parameters (-q 0.05 -g 2.7e9 --keep-dup 1). The resulting set was filtered against blacklisted chromatin regions, as detected by ENCODE. Consensus peak sets were calculated by GenomicRanges' reduce function. Read counts across peaks were determined using the featureCounts function of the Subread package. Differential binding analysis was performed by using DESeq2 after merging overlapping peaks into reference peak sets, using the reduce function of the BioConductor GenomicRanges package.

Data quality

Raw FASTQ files were inspected with fastqc. Trim_galore was used for quality aware trimming and removal of adapters using default parameters. Mapping and peak calling were evaluated by optical inspection in the genome browser as well as pair-wise correlation analysis of binding profiles (coverage).

Number of peaks > 5 fold enrichment over input and FDR<0.2:

Scramble mESCs rep1 H3K56ac 5129
Scramble mESCs rep2 H3K56ac 4803
Sirt6KD mESCs rep1 H3K56ac 10710
Sirt6KD mESCs rep2 H3K56ac 11538

MuScs_Sirt6mKO 25230
MuScs_Sirt6mKO 65110
MuScs_Sirt6mKO 92688

	MuScs_Sirt6mKO 109227

Software

FastQC (https://www.bioinformatics.babraham.ac.uk/projects/fastqc/)
Trim Galore (https://www.bioinformatics.babraham.ac.uk/projects/trim_galore/)
BWT index from Illumina's iGenome repository (https://emea.support.illumina.com/sequencing/, sequencing_software/igenome.html)
bowtie version 1.1.2
Integrative Genome Viewer (IGV),
MACS2
DESeq2
BioConductor GenomicRanges package

Flow Cytometry

Plots

Confirm that:

☒ The axis labels state the marker and fluorochrome used (e.g. CD4-FITC).

☒ The axis scales are clearly visible. Include numbers along axes only for bottom left plot of group (a 'group' is an analysis of identical markers).

☒ All plots are contour plots with outliers or pseudocolor plots.

☒ A numerical value for number of cells or percentage (with statistics) is provided.

Methodology

Sample preparation

Limb and trunk muscles were minced, digested with 100 CU Dispase (BD) and 0.2% type II collagenase (Worthington Biochemicals), and consecutively filtered through 100 µm, 70 µm, and 40 µm cell strainers (BD). Cells were collected by centrifugation at 1,200 x g for 7 minutes. Pellets were re-suspended in 1.5 ml red blood cell lysis buffer containing 5 µg/mL DNase I and incubated on ice for 3 minutes. Subsequently, the cell suspension was filled up to 7 ml with DMEM medium containing 2% FCS, before cells were spun down. To enrich for MuSCs, isolated cells were incubated with APC fluorescence coupled primary antibodies against Sca1, CD45, CD31 (1:100 dilution in FACS sorting buffer) for 40 min at 4oC. After addition of 5ml of DMEM medium containing 2% FCS, cells were spun down and the cell pellets were resuspended in 200µl FACS sorting buffer, before incubation with 30µl of anti-APC micro beads (MACS) for 15 min on 4oC. Microbeads containing Sca1+/CD45+/CD31+ cells were isolated by a 25 LS separation column using the QuadroMACS separator (Miltenyi Biotec). Sca1-/CD45-/CD31- cells were spun down, stained with Integrin-a7-FITC and CD34-Alexa Fluor 405 antibodies (1:100 dilution in FACS sorting buffer). Integrin-a7+/CD34+, GFP+ (from Pax7:ZsGreen mice) or YFP+ (from Pax7ICNCre/ROSA-YFP mice) satellite cells was isolated using a FACS AriaIII (BD Biosciences).

Instrument

FACS sorting for muscle stem cells: FACSAriaTM III (BD Biosciences)

Software

BD FACS Diva v8 software

Cell population abundance

Sorted cells were reanalyzed to assess purity. A 75-80% purity was achieved.

Gating strategy

The gating strategy identified muscle stem cells by using Integrin-a7-FITC and CD34-Alexa Fluor 405 antibodies or by taking advantage of endogenous muscle stem cell-specific fluorescence of reporter mice (Pax7:ZsGreen mice or Pax7ICNCre/ROSA-YFP mice). MuSCs population was defined using FMO (Fluorescense Minus One) FITC control (not treated with Integrin a7 – FITC) and dead cells were excluded via DAPI staining. The gating strategy is shown on Supplementary figure 9 and Supplementary figure 10.

☒ Tick this box to confirm that a figure exemplifying the gating strategy is provided in the Supplementary Information.
